# Randomness assisted in-line holography with deep learning

**DOI:** 10.1038/s41598-023-37810-w

**Published:** 2023-07-07

**Authors:** Manisha, Aditya Chandra Mandal, Mohit Rathor, Zeev Zalevsky, Rakesh Kumar Singh

**Affiliations:** 1 Laboratory of Information Photonics and Optical Metrology, Department of Physics, Indian Institute of Technology (Banaras Hindu University), Varanasi, Uttar Pradesh 221005 India; 2 Department of Mining Engineering, Indian Institute of Technology (Banaras Hindu University), Varanasi, Uttar Pradesh 221005 India; 3 Faculty of Engineering and Nano Technology Center, Bar-Ilan University, Ramat Gan, Israel

**Keywords:** Imaging and sensing, Applied optics

## Abstract

We propose and demonstrate a holographic imaging scheme exploiting random illuminations for recording hologram and then applying numerical reconstruction and twin image removal. We use an in-line holographic geometry to record the hologram in terms of the second-order correlation and apply the numerical approach to reconstruct the recorded hologram. This strategy helps to reconstruct high-quality quantitative images in comparison to the conventional holography where the hologram is recorded in the intensity rather than the second-order intensity correlation. The twin image issue of the in-line holographic scheme is resolved by an unsupervised deep learning based method using an auto-encoder scheme. Proposed learning technique leverages the main characteristic of autoencoders to perform blind single-shot hologram reconstruction, and this does not require a dataset of samples with available ground truth for training and can reconstruct the hologram solely from the captured sample. Experimental results are presented for two objects, and a comparison of the reconstruction quality is given between the conventional inline holography and the one obtained with the proposed technique.

## Introduction

Digital holography (DH) has emerged as a powerful tool for recording and reconstructing the amplitude and phase information of the wave^1–4^. The ability of the DH to retrieve complex amplitude information has a wide range of applications in 3D displays^5^, microscopy^6^, biomedical imaging^7^, and many more. The DH provides a spatially resolved quantitative phase images and depth reconstructions. In-line, off-axis, and phase shifting are a few widely used schemes. In an off-axis holography, two coherent and angularly separated beams interfere to record the hologram information^8^. Since available digital detectors have a limited pixel pitch, angular separation between interfering beams introduce a limitation for the off-axis DH. Moreover presence of unmodulated term and conjugate limits utilization of the full bandwidth in an off-axis DH geometry. Phase shifting is another technique that uses multiple recordings of the same object with phase shifts in the reference wave^9–12^. Among several holographic techniques, in-line holography has a compact design, and a high space bandwidth product (SBP)^13,14^. In-line holography schemes can be designed by using a single path and obtained by the interference of diffracted and un-diffracted waves emerging from the object^14^. However, a bottleneck of in-line holography is the ubiquitous twin image problem. Various techniques have been developed to resolve this issue using optical and computational methods^15–17^. Development of techniques such as non-interferometry and non-iterative schemes based on Kramers-Kronig method^18,19^ and dual-plane coupled phase retrieval for non-prior holographic imaging^20^ have further advanced the field of complex field imaging.

The quality of reconstruction in the DH depends on the recording configurations. Due to digital recording and constraints on the detectors, improving resolution is an emerging interest in the DH. Resolution in a digital holographic setup is influenced by factors such as; the numerical aperture, detector pitch, and diffraction. In the past, various techniques have been proposed to improve the resolution in the DH, and some of these techniques are lowering the wavelength^21^, down-sampling the detector pitch^22^, increasing the effective numerical aperture^23,24^, expanding computational bandwidth^25^. Recently, some advancements have been made to develop high SBP imaging using spatial light modulator^26^, Kramers-Kronig relation^27,28^, and off-axis digital holographic multiplexing^29^. Structured light illumination has also been used to improve the image quality and resolution^30–36^. Zheng et al. used structured illumination in different orientations combined with an iterative algorithm to enhance the spatial resolution in the DH^36^. Speckle field illumination has also been used in the DH for high-resolution imaging and in enlarging field of view^6,37–42^. However, these speckle illumination methods need recording of several holograms with the random illumination patterns for proper cancellation of randomness.

On the other hand, use of the randomness rather than cancellation has shown significant potential in imaging such as ghost imaging, ghost diffraction microscopy^43,44^, resolution enhanced wide field imaging^45^ and many more. Memory effect^46^ within a speckle pattern offers a high-resolution imaging systems^47^. Correlations have been exploited in super-resolution optical fluctuation imaging (SOFI) with dynamics near field speckle patterns^48^. It has been demonstrated that higher-order (n) intensity correlations improve the resolution by a factor of $\sqrt{n}$ and such higher-order correlations have been utilized for improving resolution beyond the diffraction limit^49,50^. A sub-Rayleigh imaging has been demonstrated using a second-order correlation measurement^51^. However, majority of these correlation techniques mainly deal with amplitude object, without any phase signature of the signal except a recent work^52^.In the reference^52^, a sub-Rayleigh dark-field imaging by speckle illumination is demonstrated and an autocorrelation image is presented for a binary phase object.

In this paper, we propose and demonstrate a new method to record an in-line hologram in intensity correlation rather than the intensity as in conventional holography. The novelty of our work lies in demonstrating enhanced complex field imaging with a better reconstruction quality. The narrower point spread function in the intensity correlations plays a critical role in enhancing the quality of reconstruction in our technique. Further, a common issue of twin images in in-line holography is tackled with an unsupervised DL method by utilizing an auto-encoder model. Based on DL, other methods have also been proposed to address this issue, such as those used in end-to-end digital hologram reconstruction^53–56^, with the capabilities of convolutional neural networks (CNNs) as a universal approximator for solving inverse problems in the computational imaging. This typically involves training a CNN on a labeled dataset such as paired holograms and corresponding twin image-free phase and amplitude data, and then use the trained CNN to reconstruct twin image-free results. However, these DL methods with CNN require large datasets for training, which can be difficult and costly to obtain in the DH. Moreover, CNNs are often considered “black boxes,” because the training and inference processes are not transparent and cannot be easily explained^17^. This can be problematic when using a well-trained CNN to reconstruct a hologram, as it is not possible to address any issue that may arise if the reconstruction is not accurate.

Therefore, in the proposed reconstruction of the inline hologram,we prefer to use an unsupervised DL approach for a single-shot image reconstruction without a large training dataset^17^.This method utilizes an autoencoder to fit the solutions of physics-driven holography equations, and similar to other applications of autoencoders, our method does not rely on a training dataset, which makes the reconstruction process easier. The auto-encoder minimizes a well-defined objective function to reduce noise and remove twin images instead of suppressing it, by adjusting its weights to find the reconstructed object that is most consistent with the captured hologram. Our results show that a neural network equipped with the proposed correlation method provides recovery of the larger spectrum and hence enhanced quality of reconstruction of the complex-valued object. Our proposed scheme is helpful in imaging beyond the diffraction limit and developing new holographic techniques. The proposed technique is experimentally verified, and reconstructed results are presented for two different cases, i.e., a conventional holographic recording and hologram recording by second-order correlation. A comparison of the conventional holography and the proposed method highlight high-quality reconstruction in the new technique. Theoretical background and experimental demonstrations are discussed below.

## Theory and methodology

**Figure 1:**
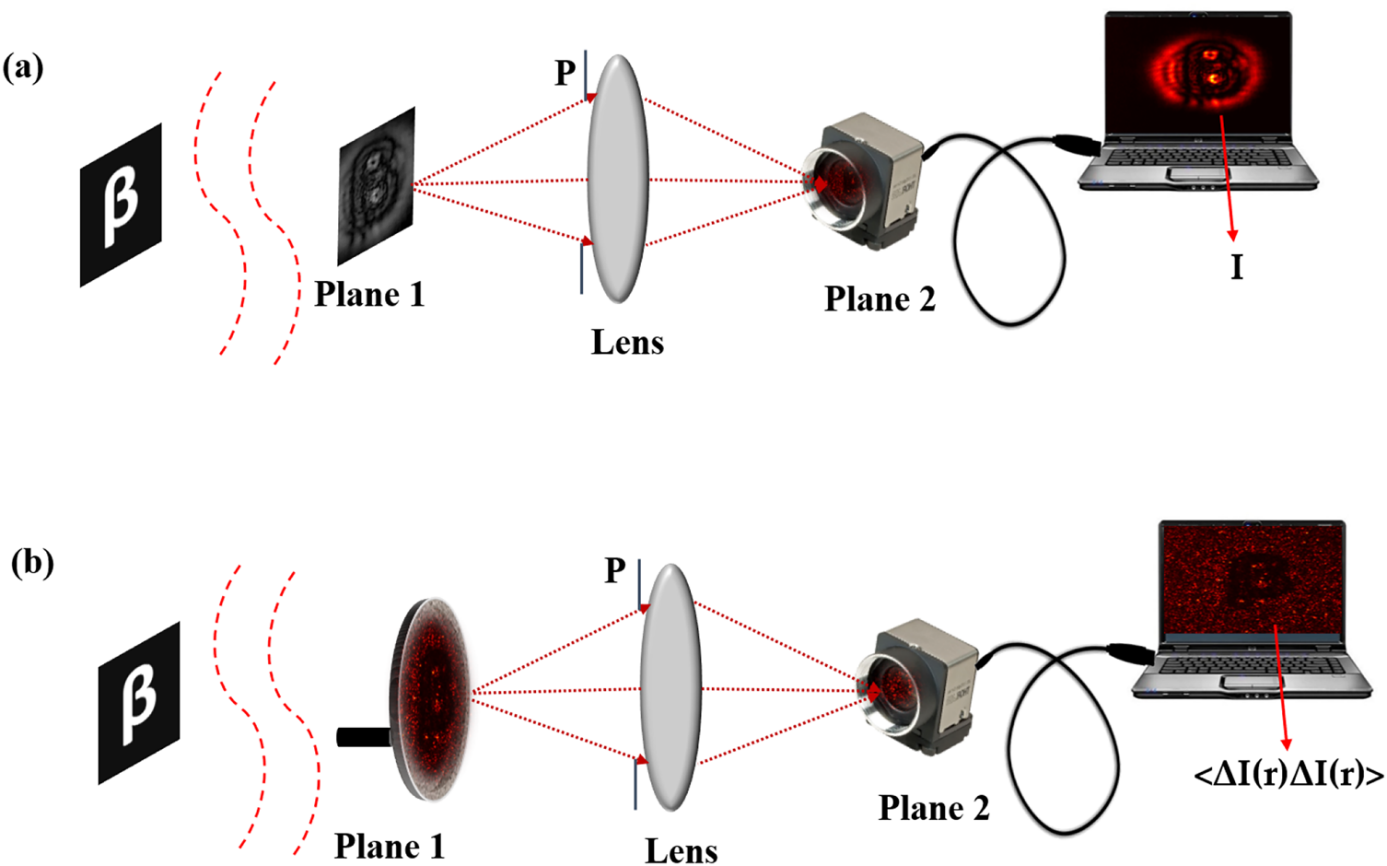
A comparison of (**a**) conventional and (**b**) proposed technique.

A conventional recording of an in-line hologram and its comparison with proposed technique is shown in Fig. 1. Diffracted and undiffracted beams from the object interfere at plane 1 and make an in-line hologram as a distribution of the intensity. This inline hologram is imaged and digitally recorded at plane 2, as shown in Fig. 1a. An aperture is placed in front of the lens to control the numerical aperture and consequently analyze its impact on the image quality. An intensity of the in-line hologram at plane 1 is given by,

1 $$\begin{aligned} I_H(\rho )=\left| E_H(\rho )\right) |^2=\left| E_D(\rho )\right| ^2 +\left| E_d(\rho )\right| ^2 +E_D^*(\rho ) E_d(\rho )+E_D(\rho ) E_d^*(\rho ) \end{aligned}$$

where $E_H(\rho )=E_{D}(\rho ) + E_{d}(\rho )$ is the complex field at plane 1. $E_{D}(\rho )$ and $E_{d}(\rho )$ are diffracted and un-diffracted beams respectively and $\rho$ is the spatial coordinate at plane 1. The optical field at plane 2 is represented as,

2 $$\begin{aligned} E(r)=\int E_H(\rho )) h(r-\rho ) d \rho \end{aligned}$$

where the digitally recorded hologram is $I=|E_H \circledast h|^2$, $\circledast$ represents the convolution operator and *r* is the spatial coordinate at plane 2. $h(r-\rho )$ represents the point spread function (PSF) of a diffraction-limited imaging system. The PSF for a diffraction-limited imaging lens is an airy disk and is represented as,

3 $$\begin{aligned} h\left( {r}, {\rho }\right) \propto \int d {\rho }_0 P\left( {\rho }_0\right) \exp \Bigg \{\frac{i \pi }{\lambda }\left[ \frac{\left( {\rho }_0-{\rho}\right) ^2}{d_1}+\frac{\left( {r}-{\rho }_0\right) ^2}{d_2}-\frac{{\rho }_0^2}{f}\right] \Bigg \} \end{aligned}$$

where P($\rho _0$) is the pupil function describing the effective entrance pupil, and $d_1$ and $d_2$ are the distances from the lens to the object plane and image plane, respectively, and $d_1=d_2=2\hbox {f}$. Equation (2) represents a digital recording of the hologram which is numerically reconstructed to recover the complex-valued object from the DH.

Now, consider a scatterer at the plane 1 which hides the direct recording of the hologram from the detector. A random scatterer in the path of the light scrambles the wavefront and generates a speckle pattern as shown in Fig. 1b.

A single realization of the field immediately after the scatterer is given by,

4 $$\begin{aligned} E(\rho ,t)=E_H(\rho ,t) \exp \left[ i \phi (\rho ,t)\right] \end{aligned}$$

where $\phi (\rho ,t)$ is the random phase introduced by the scatterer and *t* represents time corresponding to different random patterns.

The complex field at the recording plane 2 is represented as,

5 $$\begin{aligned} E(r,t)=\int E(\rho ,t)) h(r-\rho ,t) d\rho \end{aligned}$$

The random intensity at plane 2 is

6 $$\begin{aligned} I(r,t) =\left| E(r,t)\right) |^2 \end{aligned}$$

Imaging of a hologram through a scatterer has been used in the past for looking around corners^57^ and in the correlation holography^58^. These methods rely on recovering conventional hologram i.e, $I(\rho )$ from the averaged random fields. On the other hand, here we propose and experimentally demonstrate a new technique by recording the hologram in terms of the second-order correlation of the intensity rather than intensity.

Following Eq. (6), intensity fluctuation is given as,

7 $$\begin{aligned} \Delta I(r,t)= I(r,t) - \langle I(r,t)\rangle \end{aligned}$$

where angular bracket $\langle ...\rangle$ represents ensemble average and $\langle I\rangle$ is mean intensity.

We introduce and use correlation of intensity fluctuations to record the hologram and this correlation function is represented as,

8 $$\begin{aligned} g^{2}(r,r)= \langle \Delta I(r,t)\Delta I(r,t)\rangle \end{aligned}$$

Equation (8) represents our quantity of interest to record the in-line hologram. In comparison to the conventional hologram recording in I(r) , the proposed technique records hologram as a distribution of $g^{2}(r,r)$. For the Gaussian random field, second-order intensity correlation can be expressed as a modulus square of the field correlations as,

9 $$\begin{aligned} g^{2}(r,r)=|\langle E^{*}(r,t)E(r,t)\rangle |^2 \end{aligned}$$

where asterisk $*$ represents the complex conjugate. The second-order field correlation is represented as,

10 $$\begin{aligned} \langle E^{*}(r,t)E(r,t)\rangle =\iint \ E^{*}(\rho _1)E(\rho _2)\langle exp (-i \phi (\rho _1,t))\exp \left( i \phi (\rho _2,t)\right\rangle _T\ h^{*}(r-\rho _1)h(r-\rho _2) d\rho _1 d\rho _2 \end{aligned}$$

For a spatially incoherent source, the correlation at plane 1 is represented as,

11 $$\begin{aligned} \langle \exp i(( \phi (\rho _2,t)-\phi (\rho _1,t))\rangle _{T}=\delta (\rho _2-\rho _1) \end{aligned}$$

Substituting Eq. (11) in Eq. (10) leads to

12 $$\begin{aligned} \langle E^{*}(r,t)E(r,t)\rangle =I(\rho ) \circledast h^{2}(r-\rho ) \end{aligned}$$

Therefore second-order intensity correlation is expressed as,

13 $$\begin{aligned} g^{2}(r,r)=\left| I \circledast h^{2}\right| ^2 \end{aligned}$$

For a uniform source, i.e, $I(\rho )=1$; the second order intensity correlation transforms to,

14 $$\begin{aligned} g^2\propto h^4 \end{aligned}$$

Equation (14) represents that the correlation of intensity fluctuations is proportional to the fourth power of the PSF of an imaging lens. A comparison of Eqs. (2) and (14) shows that the recording quality is enhanced in the higher-order correlation due to a narrower size of the PSF as compared to the conventional case. The size of the Airy disk is reduced by a factor of 0.6 in the measurement of the intensity correlation $g^2 (r,r)$ when compared to the intensity measurement^51^. The proposed scheme is quantitatively analyzed and experimentally tested in the coming section. Although the quality of recording the inline hologram is improved in the randomness-assisted approach, reconstruction of an inline hologram demands the removal of the twin images. This is tackled by using an unsupervised DL method as explained in the coming section.

## Experimental recording

### Experimental recording and numerical reconstruction of inline hologram

**Figure 2:**
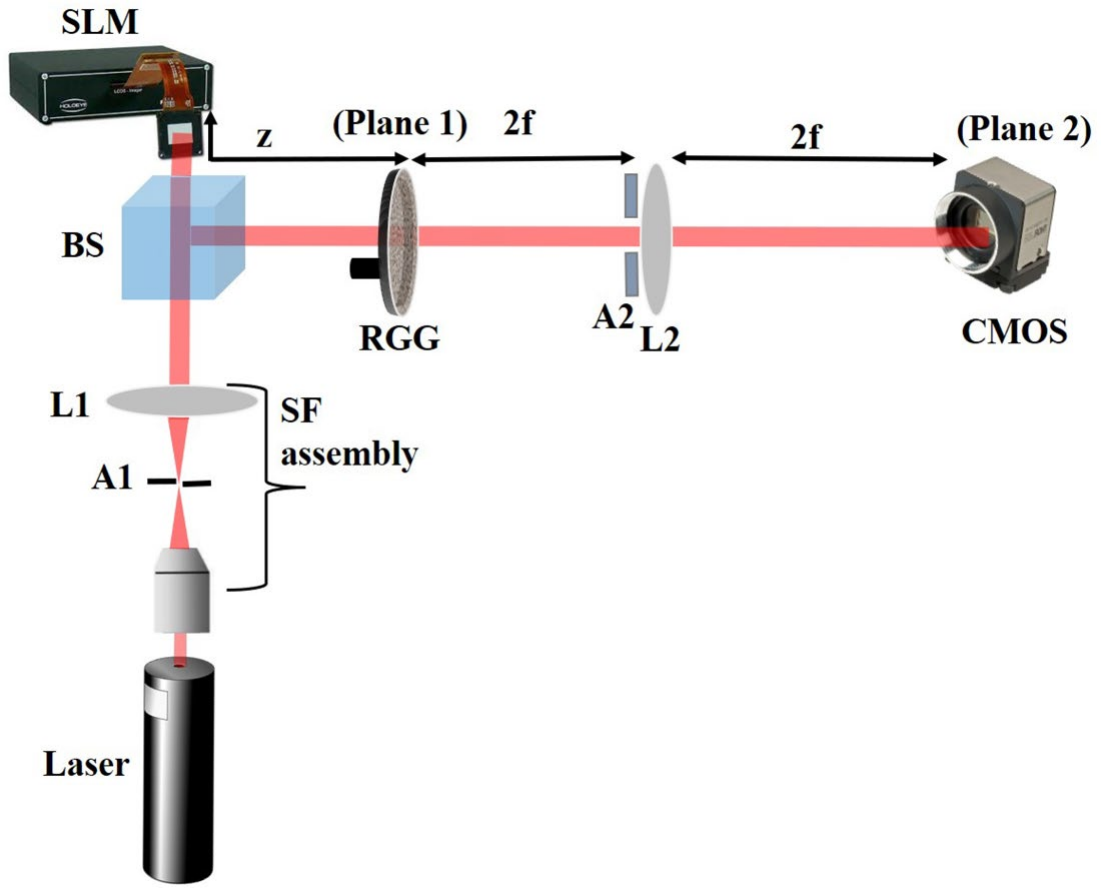
Experimental Setup; SF: Spatial filter, L1 and L2:Lens with focal lengths 200 and 100 mm respectively, BS: Beam splitter, SLM: Spatial light modulator, RGG: Rotating ground glass, A1 and A2:Apertures, CMOS Camera: Complementary metal oxide semiconductor camera.

A monochromatic laser beam of wavelength 633 nm (Thorlabs, Model No. HNL 150L) is spatially filtered and collimated by a spatial filter assembly, and a lens L1. A beam splitter (BS) divides the incident beam into two equal reflected and transmitted components. The beam transmitted from BS is used to illuminate a reflective type spatial light modulator (SLM). This SLM is having $1280\times 768$ pixels with a pixel pitch of $20\,\upmu \hbox {m}$. An object is inserted in the incident light using the SLM and the light reflected from the SLM is folded by the BS to propagate toward the plane 1 (without rotating ground glass (RGG)). The distance between SLM and plane 1 is 200 mm. Interference of diffracted and undiffracted waves from the object makes an inline hologram at the plane 1 which is imaged by lens L2 at the camera plane using a 4f imaging system. Focal length of lens L2 is 100 mm and a variable aperture A2 is placed to control the numerical aperture of the imaging lens as shown in Fig. 2. A CMOS camera with $1280\times 1024$ pixels, and pixel size 5.3 μm, records the in-line hologram.

In order to replace conventional hologram recording by recording in the correlation of intensity fluctuations, we insert a RGG at plane 1 in the experimental system. An inline hologram at plane 1 is now scrambled due to the random phase introduced by the RGG. Therefore recorded pattern at the camera plane represents a speckle pattern for a particular position of the RGG and the pattern is represented as $| E \circledast h|^2$. This random phase is common to both the diffracted and undiffracted beams at the plane 1, thus hologram information can be retrieved by averaging the random patterns in spite of randomness in the path of the light. The randomly scattered inline hologram is recorded using different orientations of the RGG at different times t. For experimental demonstrations of the proposed technique, we used 150 frames which are independently generated by the RGG. A series of random patterns with independent randomness are utilized to measure the correlation of the intensity fluctuations and to record the inline hologram. In the randomness-assisted recording of an inline hologram, a correlation of the intensity fluctuations is measured by estimating correlations of intensity fluctuations over varying random patterns. We considered averaging over 150 random patterns and is represented as,

$$\begin{aligned} \sum _{n=1}^{\ 150}\left[ I_n(r) -\langle \ I(r) \right\rangle ]\left[ I_n(r) -\langle \ I(r) \right\rangle ] \end{aligned}$$

This equation represents the correlation of the fluctuations of intensity patterns corresponding to 150 independent random patterns generated by the ground glass. Each shot of the recorded image has a noisy pattern for fixed orientation of RGG at a time t. We make use of averaging over a number of random patterns and $g^2(r,r)$ is estimated. This results in a hologram with reduced noise and an inline hologram appearing as a distribution of $g^2(r,r)$.

**Figure 3:**
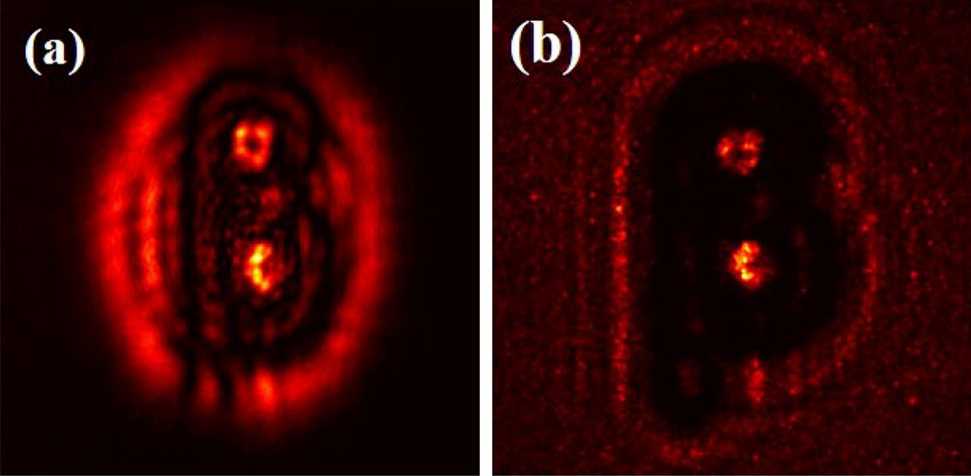
(**a**) Conventional inline hologram for $\beta$; (**b**) Inline hologram in terms of second order correlation.

Once we have the recorded hologram, we digitally reconstruct it to obtain the quantitative amplitude and phase distribution. Quality of the reconstruction is influenced by the total number of incoherent random patterns^59^. Moreover, the object needs to be static during the recording of the random patterns. The spatial resolution achieved in the proposed scheme is also influenced by the finite correlation length of the random phase screen employed in the experiment, which imposes a cap on the hologram’s spatial fineness. We assume that the random screen is delta correlated, and any deviation from it would lead to degraded reconstruction quality of the hologram. The experiment is performed for two different objects, a Greek letter “$\beta$” and an English letter “*V*” . In order to test and prove the validity of the proposed scheme, we used two quantitative objects with sharp edges and curved structures. These objects were selected to examine the role of the high-frequency content in image quality reconstruction and quantitative evaluation of the performance of the proposed scheme with higher-order correlation by comparing it with conventional schemes. Objects, such as “$\beta$” of size ($2.8~\text {mm} \times 1.48~\text {mm}$) and $V(2.84~\text {mm}\times 2.64~\text {mm})$ were used in the experiment with the help of SLM. An inline hologram of letter $\beta$ is shown in Fig. 3a recorded as a distribution of the intensity *I*(*r*) and recording of an inline hologram of the same object as a distribution of second order intensity correlation $g^2(r,r)$ is represented in Fig. 3b. The detailed procedure of retrieving quantitative information from the inline hologram with and without randomness is described below.

### Deep learning for numerical reconstruction of inline hologram

In the past, different methods have been proposed to tackle reconstruction of the in-line hologram, including the phase retrieval algorithms^60–65^. Accurate recovery of the phase can lead to strong suppression of the twin image^66,67^. We use an unsupervised DL approach for reconstructing the in-line hologram using an encoder–decoder architecture^17^. This network maps a high dimensional input image, $\alpha$, into a low dimensional latent code, $\beta$. The mapping from the input image to the latent code and the reconstruction of the output from the latent code are represented by the functions $F_{\text {encode}}(\alpha ) = \beta$ and $F_{\text {decode}}(\beta ) = {\hat{\alpha }}$, respectively. This model can learn the uncorrupted and realistic features of the input image through an iterative process, allowing it to converge on a denoised output. To address the twin image issue in an inline hologram, a physical model is designed for training the auto-encoder. The auto-encoder, denoted as $F_{auto-encoder}(\alpha , w)$, is trained to minimize the error between the captured in-line hologram *H*_g_(*I*(*r*) or $g^2(r,r)$ ) and the reconstructed in-line hologram, which is the forward-propagated result of the reconstructed complex object wave to the plane 1 using $F_{transmission}$. The weights *w* are randomly initialized at the start of training. The goal is to find the weights *w* that minimize the error between the captured in-line hologram and the reconstructed in-line hologram. The objective function can be written as:

15 $$\begin{aligned} w={\arg \min }|H_{g}-F_{transmission}(F_{auto-encoder}(H_{g}, w))|_2^2 \end{aligned}$$

Where $|. |_{2}$ is $L_2$ norm. The in-line hologram obtained from Eq. (14) is first back-propagated to the object plane to determine the amplitude and phase distributions. These are then fed into the auto-encoder model. The network outputs the reconstructed amplitude and phase, which are then forward propagated to the in-line hologram plane using $F_{transmission}$ to get a reconstructed in-line hologram. The mean absolute error (MAE) loss between the original hologram and reconstructed hologram is calculated to update the model’s weights.

### Description of auto encoder model

**Figure 4:**
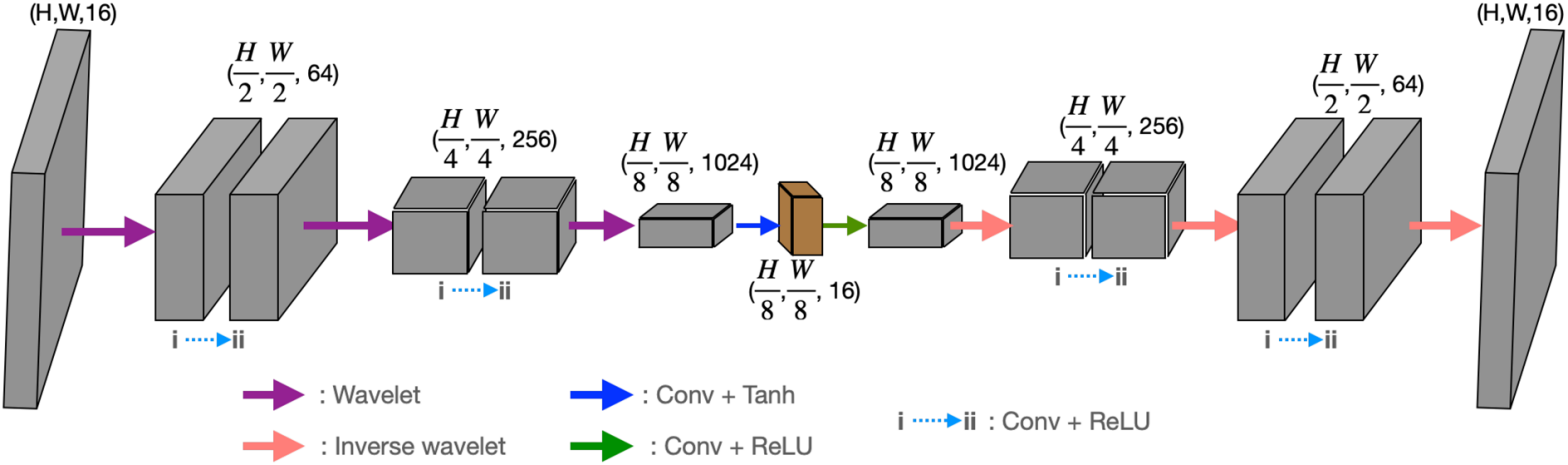
An auto-encoder network utilizing deep convolutional layers in an hourglass architecture.

Here, we use an “Hourglass” type auto-encoder architecture. This encoder transfers the fixed network input into lower-dimensional space; encoding steps, and decoding steps are done by reconstructing the complex object from their representation by the latent variables. To get better convergence of our model, we use batch normalization^68^ and ReLU activation^69^ function after most layers. In this network architecture, we use a wavelet transform and its inverse transform to provide downsampling (encoding) and upsampling (decoding), which substitute strided convolution, pooling, or interpolation. In this case, the encoding and decoding processes make use of the 2D Haar wavelet and its inverse transform^17^. We found that skip connection (similar to the U-Net^70^) is not useful for our proposed case since the network will directly project the input to the output rather than learning a clear reconstruction of the complex field and the same notion applies when the number of channels after encoding is much higher. Skip connection maybe useful in conventional image restoration but not for phase retrieval case. To resolve this issue, encoder’s feature channels at the output is compressed from 1024 into 16 channels, as indicated by the blue arrow in Fig. 4. The input and output image shapes for the auto-encoder network are (H, W, 2), where H and W represent the height and width of the image, respectively. The two channels correspond to the amplitude and phase of the back-propagated in-line hologram.

Through iterations, it was found that the network first generates a rough estimate of the reconstructed object, and then gradually recovers the details of the object. When the objective function is minimized using the gradient descent method, the network is able to find the optimal results by adjusting the model parameters within the parameter space. This is done by generating a rough estimate of the reconstructed complex object, known as the primary instance, and then progressively refining this estimate through the recovery of the twin image free amplitude, and phase details of the complex object from coarse to fine until the final reconstructed complex object is obtained. In this manner, the network tunes the model parameters within the parameter space to search for optimal results.

## Results and discussion

**Figure 5:**
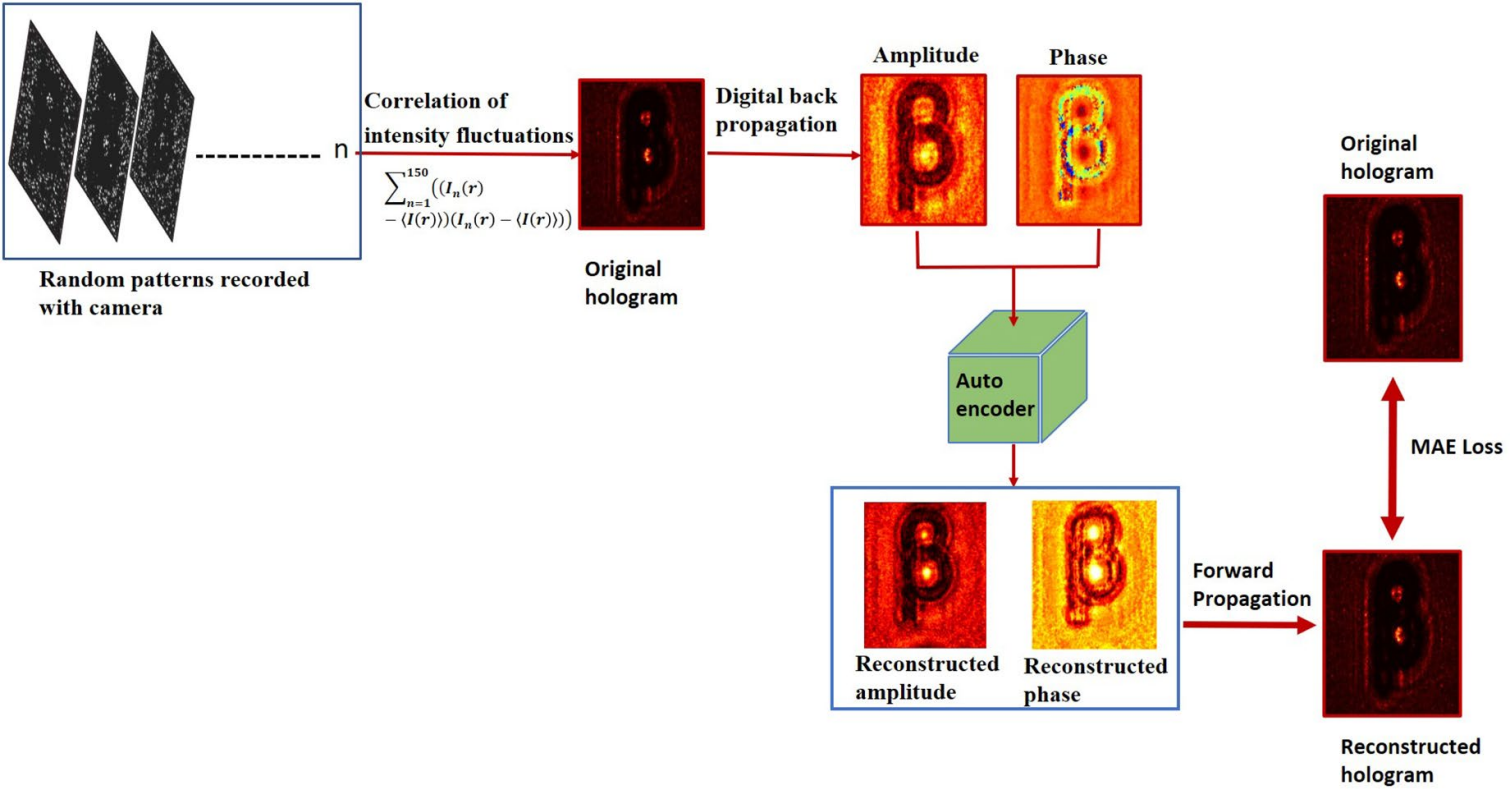
A flow chart for the proposed scheme.

**Figure 6:**
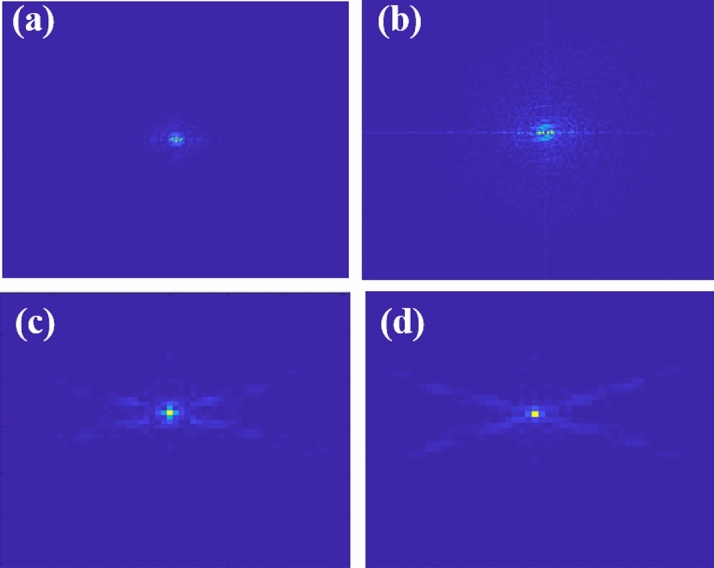
(**a**, **b**) Fourier spectrum for $\beta$ in conventional case and proposed scheme respectively; (**c**, **d**) Fourier spectrum for *V* in conventional case and proposed scheme respectively.

**Figure 7:**
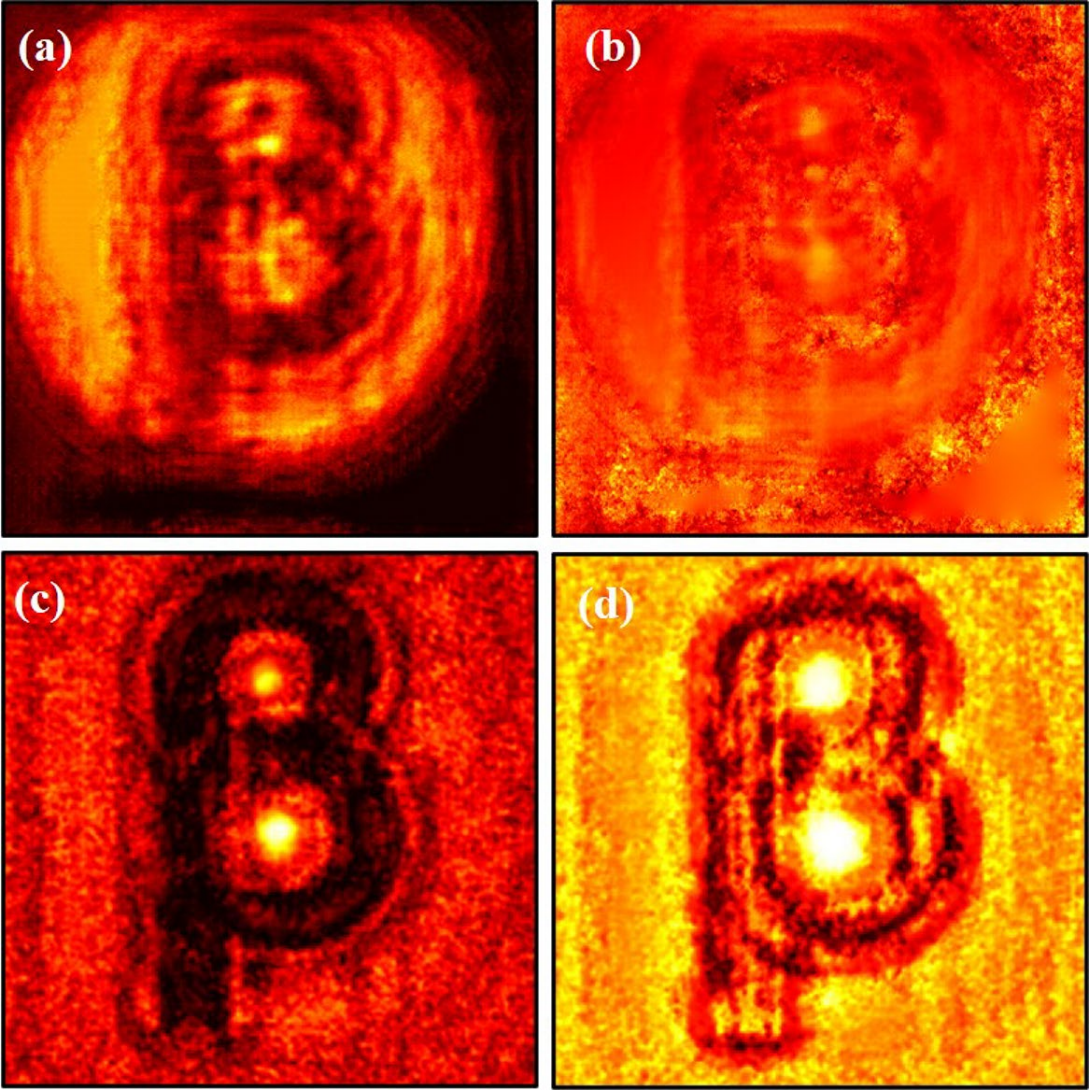
(**a**, **b**) Conventional case results for letter $\beta$; (**a**) Amplitude (**b**) Phase, (**c**, **d**) Proposed scheme based results for letter $\beta$; (**c**) Amplitude (**d**) Phase.

**Figure 8:**
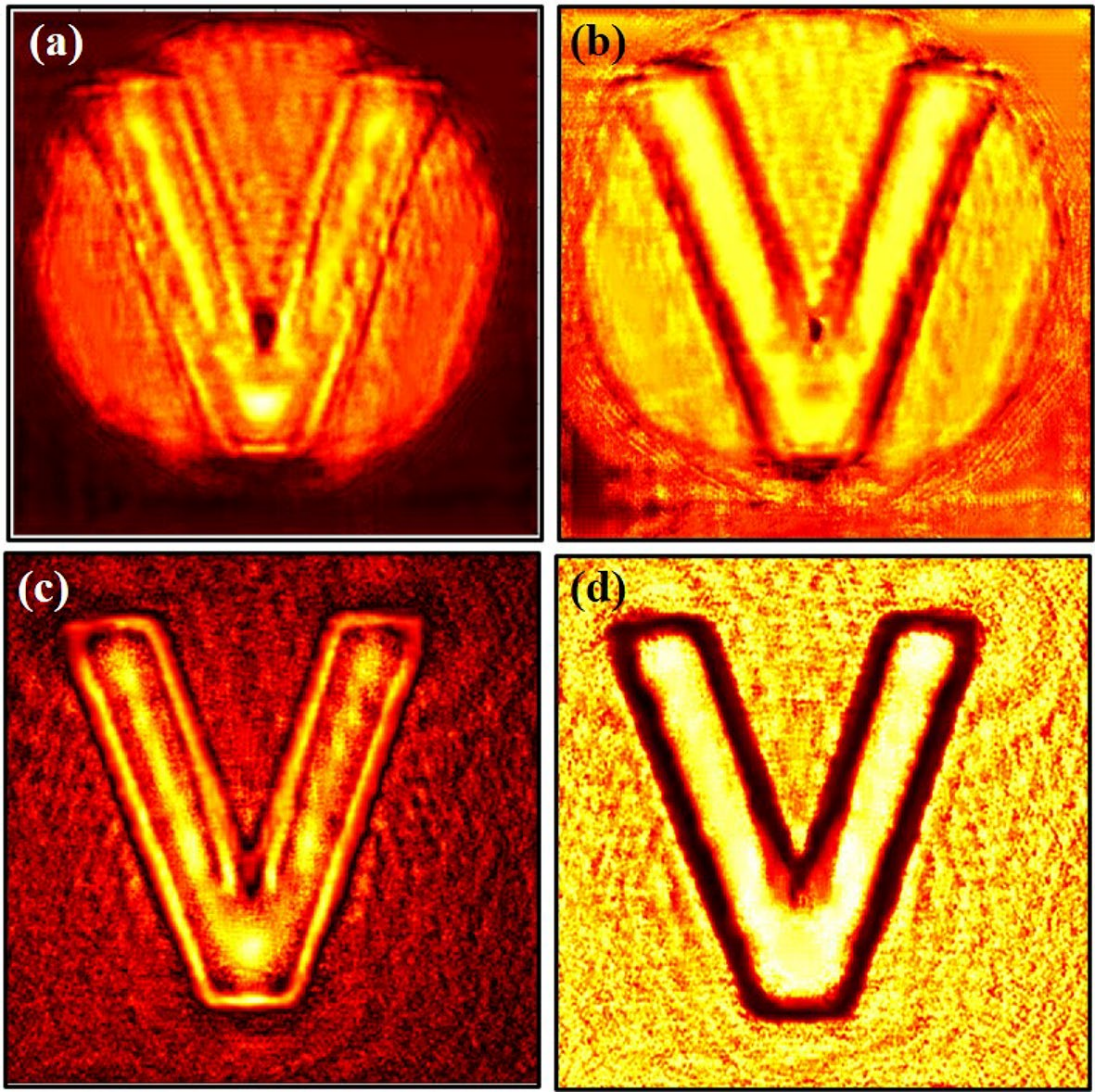
(**a**, **b**) Conventional case results for letter V; (**a**) Amplitude (**b**) Phase, (**c**, **d**) Proposed scheme-based results for letter V; (**c**) Amplitude (**d**) Phase.

We record inline holograms in two different experimental configurations, as shown in Fig. 1. In the first case, an inline hologram is recorded in free space i.e., without a scatterer. The inline hologram at plane 1 is imaged by a lens L2 with a variable aperture A2 in front of the lens L2. In the second case, when a scatterer is placed at plane 1, a speckle is generated due to the randomness and interference of randomly scattered waves. Light is scrambled into the speckle pattern without any direct resemblance with the hologram I(r). We recorded the data for the conventional and proposed scheme for a fixed aperture of diameter 2.6 mm as shown in experimental Fig. 2. For randomness-assisted inline hologram recording, a series of random patterns are captured and these random patterns are digitally processed to obtain the second-order correlation of intensity. The inline hologram is back-propagated to the object plane by using an angular spectrum method. However, inline configuration of the recording introduces a twin image in the reconstruction. Therefore a deep learning is applied to quantitatively reconstruct the inline hologram without a twin image. We employ the auto-encoder model to resolve this problem and this is implemented using the PyTorch framework and runs on a GPU workstation with an Nvidia tesla P100 graphics card. The Adam optimization algorithm is utilized with a fixed learning rate of 0.005. To train the network, we used angular spectrum back-propagation reconstruction of the in-line hologram as input and use the training process for 4000 iterations until we get the final reconstruction. The entire training process takes approximately 731 s to complete. A flow chart for the process is given in Fig. 5. To prove the validity of the proposed scheme for enhanced quality reconstruction over the conventional scheme, we evaluated the Fourier spectrum of the reconstructed objects. Fig. 6a and b represent two Fourier spectrums corresponding to an object, the letter $\beta$, for the conventional and proposed scheme. Fig. 6c and d represent two Fourier spectrums corresponding to an object, the letter *V*, for the conventional and proposed scheme. Reconstruction of an object with sharper edges exhibits higher frequency content in its Fourier spectrum. Fig. 6b and d clearly show an enlargement in the Fourier spectrum in comparison to Fig. 6a and c, respectively, and thus a better quality reconstruction is obtained with the use of hologram recording in terms of the intensity correlation. Reconstruction results are presented for two different objects viz, $\beta$ and *V* as shown in Figs. 7 and 8, respectively, in free space and randomness-assisted geometry. Fig. 7a and b are the amplitude and phase of letter $\beta$ in the inline holography without scatterer. Fig. 7c and d represent the retrieved amplitude and phase for the randomness-assisted hologram recording. Fig. 8a and b are the amplitude and phase for letter V in the inline holography without scatterer, and Fig. 8c and d show reconstructed amplitude and phase distribution for the proposed randomness-assisted inline holography technique. Comparison of inline holography in free space and through randomness shows that reconstruction quality is significantly enhanced in the hologram recording with the second-order intensity correlation.

**Figure 9:**
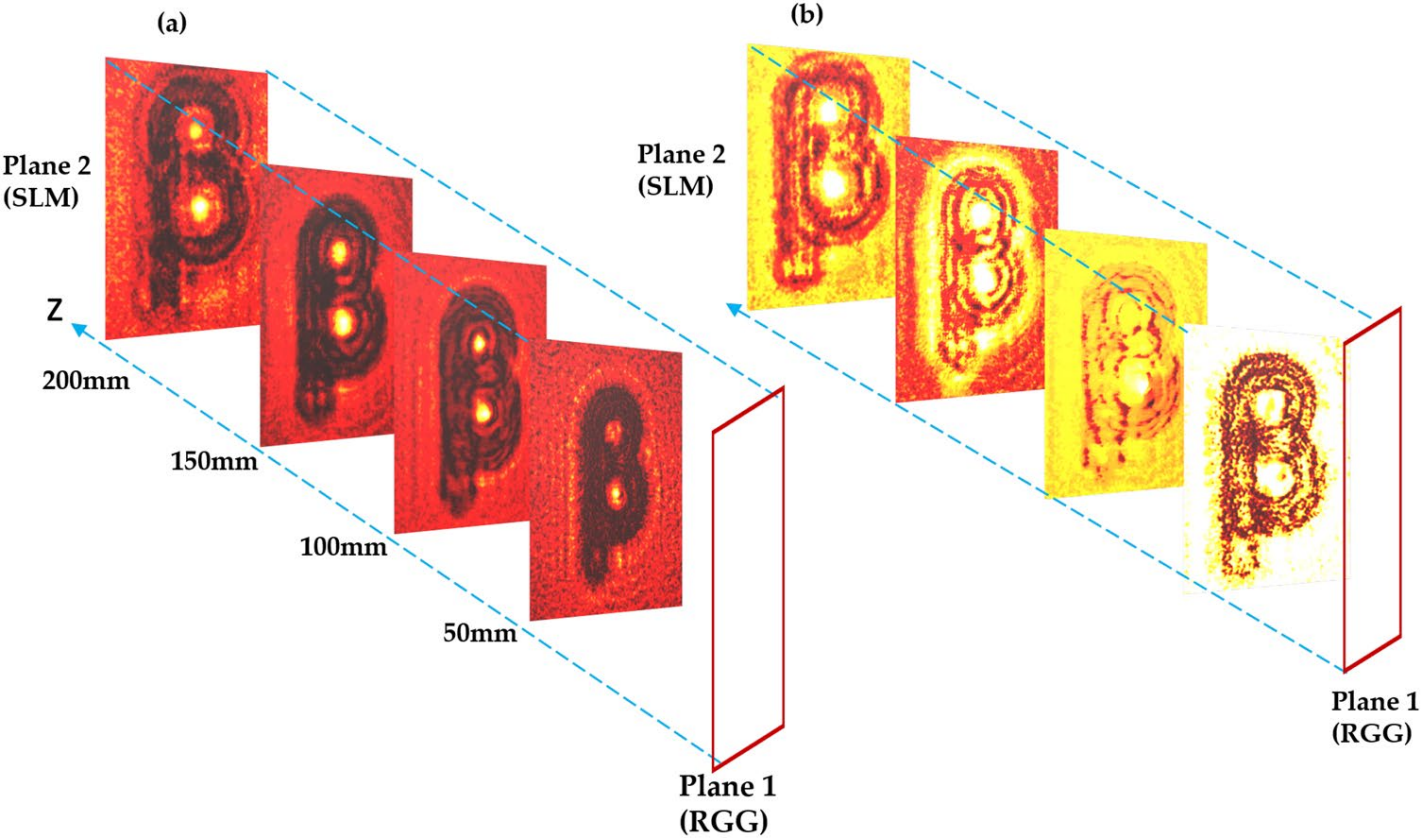
Effect of propagation: (**a**) Amplitude distribution at different propagation distances ; (**b**) Phase distribution at different propagation distances.

In order to check the reconstruction performance at various z-planes behind plane 1 and examine the effect of propagation at different distances, we have recovered the complex fields at various z planes behind plane 1 ranging from $z=50$ to 200 mm with an interval of 50 mm using digital propagation. Figures 9a and b represent the effect of propagation on amplitude and phase distribution, respectively, for propagation distances $z= 50,100,150$ and 200 mm behind plane 1.

We evaluated the performance and effectiveness of the proposed method for two different objects, i.e., for $\beta$ and *V*. The performance of the technique is quantitatively evaluated and compared with the conventional holographic reconstruction results. First, we compare the quality of the spectrum recovery, where we notice that the recovered spectrum in the proposed scheme is larger in comparison to the conventional scheme, as shown in Fig. 6. and this enhancement in the spectrum recovery also appears in the quality of reconstruction in the proposed scheme when compared to the conventional scheme. Edges and frequency contents are enhanced in the holography with $g^2(r,r)$ in comparison to the holography with *I*(*r*). This is observed by accessing the spatial frequency contents of the reconstructed object for both inline holographic recording methods, i.e., *I*(*r*) and $g^2(r,r)$. Apart from the spectrum, we also quantitatively evaluate the results using two quantitative parameters and definitions.

**Figure 10:**
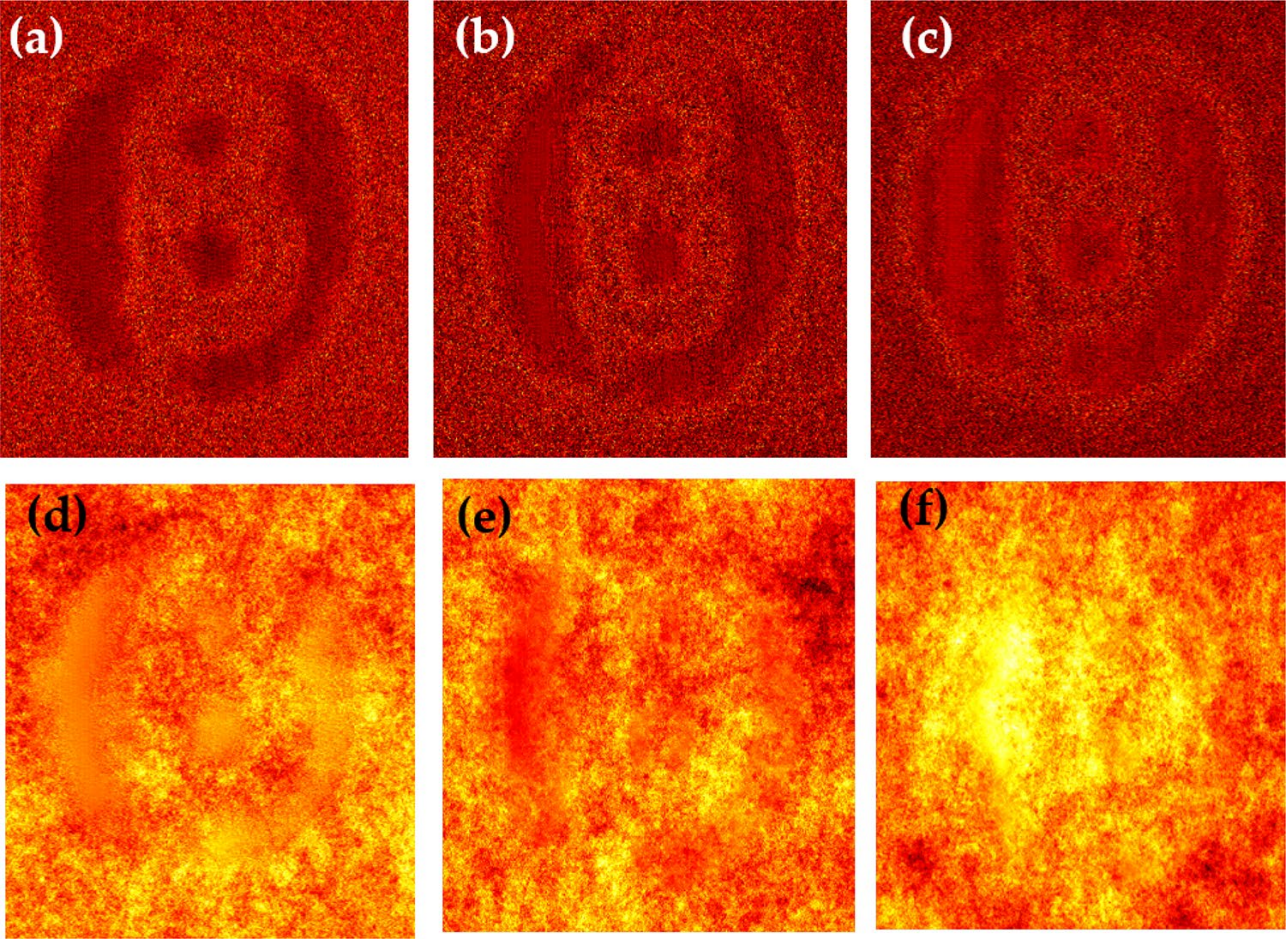
Conventional scheme: Reconstruction under different noise levels: (**a**–**c**) represent reconstructed amplitude under noise level 3,4 and 5 respectively; (**d**–**f**) represent reconstructed phase under noise level 3,4 and 5 respectively.

**Figure 11:**
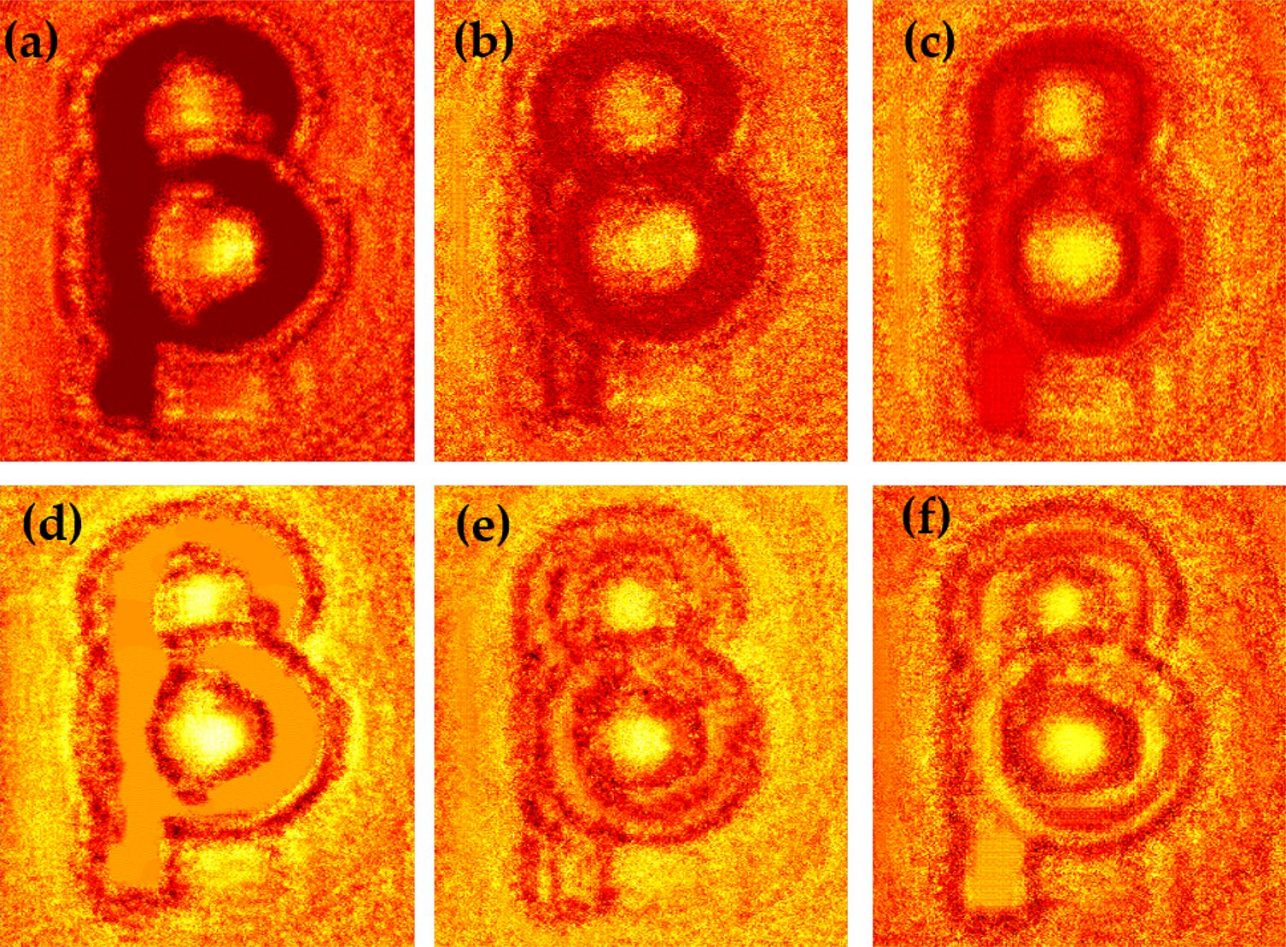
Proposed scheme: Reconstruction under different noise levels:(**a**–**c**) represent reconstructed amplitude under noise level 3, 4 and 5 respectively; (**d**–**f**) represent reconstructed phase under noise level 3, 4 and 5 respectively.

For a quantitative evaluation and comparison of the quality of reconstruction of both hologram recording methods, we introduce two parameters, such as visibility and reconstruction efficiency^71^. Visibility gives a measure of the degree to which the signal reconstruction can be differentiated from the background noise. It is defined as the ratio of the signal region’s average intensity level to the background region’s average intensity level in the reconstructed image. On the other hand, reconstruction efficiency ($\eta$) is the proportion of the signal region’s measured power to the total of the signal and background regions’ measured powers in the reconstructed image. A promising higher value of visibility and reconstruction efficiency proves the validity of the enhanced quality reconstruction. Visibility values for letter $\beta$ in the conventional and proposed scheme are 0.042 and 0.21, whereas respective reconstruction efficiency values are 0.55 and 0.885. For other object V, visibility and reconstruction efficiency values are 0.081 and 0.67 for the conventional case. The improved visibility and reconstruction efficiency values for object V in the proposed scheme are 0.55 and 0.92. For the proposed scheme, visibility for letter $\beta$ is almost five times of conventional reconstruction and is around 6.8 times for letter *V*. Additionally, we have also evaluated the Fourier spectrum corresponding to the conventional and proposed scheme, and an enlarged Fourier spectrum obtained in the proposed scheme (Figs. 6b and d) also appears in an enhanced quality reconstruction of the objects, as shown in Figs. 7c,d and 8c,d. Furthermore, we have evaluated the visibility and reconstruction efficiency at different z planes to quantitatively verify the effect of propagation in the system, as shown in Table 1. Moreover, to check the robustness of our proposed scheme, we investigate the performance of the technique when the experimentally recorded hologram is added with artificially generated additive white Gaussian noise. We add noise (variance) in the experimentally recorded hologram and check its reconstruction quality for the conventional case. On the other hand, in the proposed method, the noise is added in each recorded speckle pattern, and then the intensity correlation is applied to retrieve the inline hologram from the randomness. Figure 10 shows the reconstruction of amplitude (a–c) and phase (d–f) for three different noise levels at 3,4 and 5, respectively, in the conventional scheme. Figure 11 shows the reconstruction of amplitude (a–c) and phase (d–f) for noise levels at 3,4, and 5, respectively, for our proposed scheme. A comparison of Figs. 10 and 11 demonstrate that the proposed method holds a better performance than the conventional method when encountering noise in the recorded holograms. Table 2 gives the values of visibility and reconstruction efficiency at different noise levels for the proposed scheme. We would like to point out that the reconstruction quality also depends on factors such as the delta correlation of the random phase screen in the experimental condition and the number of incoherent random patterns in the recording of the hologram.

**Table 1:** Visibility and Reconstruction efficiency values at different z planes.

Distance from Plane 1 (mm)	Visibility	Reconstruction efficiency ($\eta$)
200	0.21	0.885
150	0.201	0.68
100	0.187	0.55
50	0.12	0.14

**Table 2:** Visibility and reconstruction efficiency values for the proposed scheme at different noise levels.

Noise level (Variance)	Visibility	Reconstruction efficiency ($\eta$)
0	0.21	0.885
3	0.19	0.72
4	0.16	0.40
5	0.13	0.25

## Conclusion

In conclusion, we have demonstrated a new method to record holograms in the second-order intensity correlation, and the advantage of this recording appears in the quality of the reconstructed object from the hologram. In order to demonstrate the proposed scheme of recording in the holography, we recorded inline holograms in both conventional and randomness-assisted approaches, and the results are compared. An enlarged Fourier spectra corresponding to the reconstructed field in the proposed scheme validates the enhanced quality imaging in comparison to the conventional holographic scheme. A twin image issue of the inline hologram is resolved with a deep learning method that uses an auto-encoder model. The quantitative features are retrieved, and quality is compared in the conventional and the proposed schemes. Moreover, the robustness of the scheme is tested and quantitatively examined by adding simulated white Gaussian noise. The scheme is expected to be useful in imaging beyond the diffraction limit and developing new holographic schemes.

## Data Availability

The data that support the findings of this study are available on reasonable request from the corresponding author.
